# Division of labor and cooperation between different butyrophilin proteins controls phosphoantigen-mediated activation of human γδ T cells

**DOI:** 10.21203/rs.3.rs-2583246/v1

**Published:** 2023-02-15

**Authors:** Mohindar Murugesh Karunakaran, Hariharan Subramanian, Yiming Jin, Fiyaz Mohammed, Brigitte Kimmel, Claudia Juraske, Lisa Starick, Anna Nöhren, Nora Länder, Carrie R. Willcox, Rohit Singh, Wolfgang W. Schamel, Viacheslav O. Nikolaev, Volker Kunzmann, Andrew J. Wiemer, Benjamin E. Willcox, Thomas Herrmann

**Affiliations:** Institute for Virology und Immunobiology, University of Würzburg, Würzburg, Germany; Institute of Experimental Cardiovascular Research, University Medical Center Hamburg-Eppendorf, Hamburg, Germany. DZHK (German Centre for Cardiovascular Research), partner site Hamburg/Kiel/Lübeck, Germany; Department of Pharmaceutical Sciences, University of Connecticut, Storrs, CT 06269, USA; Institute for Systems Genomics, University of Connecticut, Storrs, CT 06269, USA; Institute of Immunology and Immunotherapy, University of Birmingham, UK; University Hospital Wuerzburg, Department of Internal Medicine II and Comprehensive Cancer Center (CCC) Mainfranken Wuerzburg, Wuerzburg Germany; Signaling Research Centers BIOSS and CIBSS and Department of Immunology, Faculty of Biology, University of Freiburg, Freiburg, Germany; Centre for Chronic Immunodeficiency (CCI), Faculty of Medicine, University of Freiburg; Spemann Graduate School of Biology and Medicine (SGBM), University of Freiburg, Freiburg, Germany; Institute for Virology und Immunobiology, University of Würzburg, Würzburg, Germany; Institute for Virology und Immunobiology, University of Würzburg, Würzburg, Germany; Institute for Virology und Immunobiology, University of Würzburg, Würzburg, Germany; Institute of Immunology and Immunotherapy, University of Birmingham, UK; Department of Pharmaceutical Sciences, University of Connecticut, Storrs, CT 06269, USA; Institute for Systems Genomics, University of Connecticut, Storrs, CT 06269, USA; Signaling Research Centers BIOSS and CIBSS and Department of Immunology, Faculty of Biology, University of Freiburg, Freiburg, Germany; Centre for Chronic Immunodeficiency (CCI), Faculty of Medicine, University of Freiburg; Spemann Graduate School of Biology and Medicine (SGBM), University of Freiburg, Freiburg, Germany; Institute of Experimental Cardiovascular Research, University Medical Center Hamburg-Eppendorf, Hamburg, Germany. DZHK (German Centre for Cardiovascular Research), partner site Hamburg/Kiel/Lübeck, Germany; University Hospital Wuerzburg, Department of Internal Medicine II and Comprehensive Cancer Center (CCC) Mainfranken Wuerzburg, Wuerzburg Germany; Department of Pharmaceutical Sciences, University of Connecticut, Storrs, CT 06269, USA; Institute for Systems Genomics, University of Connecticut, Storrs, CT 06269, USA; 6Institute of Immunology and Immunotherapy, University of Birmingham, UK; Institute for Virology und Immunobiology, University of Würzburg, Würzburg, Germany

**Keywords:** Butyrophilin, phosphoantigens, BTN3A1, BTN2A1, Vγ9Vδ2 T cells, γδ T cells, TCR, juxtamembrane

## Abstract

Butyrophilin (BTN)-3A and BTN2A1 molecules control TCR-mediated activation of human Vγ9Vδ2 T-cells triggered by phosphoantigens (PAg) from microbes and tumors, but the molecular rules governing antigen sensing are unknown. Here we establish three mechanistic principles of PAg-action. Firstly, in humans, following PAg binding to the BTN3A1-B30.2 domain, Vγ9Vδ2 TCR triggering involves the V-domain of BTN3A2/BTN3A3. Moreover, PAg/B30.2 interaction, and the critical γδ-T-cell-activating V-domain, localize to different molecules. Secondly, this distinct topology as well as intracellular trafficking and conformation of BTN3A heteromers or ancestral-like BTN3A homomers are controlled by molecular interactions of the BTN3 juxtamembrane region. Finally, the ability of PAg not simply to bind BTN3A-B30.2, but to promote its subsequent interaction with the BTN2A1-B30.2 domain, is essential for T-cell activation. Defining these determinants of cooperation and division of labor in BTN proteins deepens understanding of PAg sensing and elucidates a mode of action potentially applicable to other BTN/BTNL family members.

## Introduction

Vγ9Vδ2 T cells comprise 1–5% of human peripheral blood T cells. They are massively expanded in some infections and exert multiple effector functions such as perforin-mediated cell lysis, help for other immune cells and peptide antigen-presentation. These functions are instrumental in the control of infection and tumors. Consequently, they have become the subject of an increasing number of preclinical and clinical studies ^1–3^

Vγ9Vδ2 TCRs contain a semi-invariant γ chain with a Vγ9JP (alternatively termed Vγ2Jγ1.2) rearrangement and highly diverse Vδ2-bearing δ chains ^4^ and are activated by diphosphorylated isoprenoid metabolites (phosphoantigens, or PAgs) such as host-derived isopentenyl diphosphate (IPP) and microbially derived (*E*)-4-hydroxy-3-methyl-but-2-enyl diphosphate (HMBPP). In some tumors and infected cells, IPP levels reach a level sufficient to activate Vγ9Vδ2 T cells ^5–8^. This activation can also be achieved pharmacologically by aminobisphosphonates (e.g. zoledronate), which inhibit the IPP-catabolizing farnesyl disphosphate synthase ^5,9^ or by farnesyl diphosphate synthase specific inhibitory RNA ^10^. HMBPP is the immediate precursor of IPP in the non-mevalonate pathway of IPP synthesis in many eubacteria, in apicomplexan parasites such as *Plasmodium spp*., and in chloroplasts. PAg-activity of HMBPP is several orders of magnitude higher than that of IPP ^11,12^.

PAg-mediated activation of Vγ9Vδ2 T cells requires expression of butyrophilin 2A1 (BTN2A1) ^13,14^ and butyrophilin 3A1 (BTN3A1) ^15^ by the stimulator or target cell. Both molecules are single membrane-spanning type I proteins composed of a B7-like extracellular region comprising an N-terminal IgV-like (V) and a membrane-proximal IgC-like (C) domain, a transmembrane domain, and a cytoplasmic region comprising a juxtamembrane (JM) region and a B30.2 domain ^16,17^. BTN2A1 binds with its V-domain to germ-line encoded regions in the CDR2 and HV4 regions of the Vγ9-domain of the TCRγ chain ^13,14^ and to the V domain of BTN3A1. The BTN3A1-B30.2 domain binds to PAg ^18 19^. Furthermore, we and others showed that the binding of PAg to the B30.2 domain of BTN3A1 induces binding of the latter to the B30.2 domain of BTN2A1 ^20 21^, a process in which the JM regions of both molecules play a pivotal role. How these events finally translate into TCR-mediated Vγ9Vδ2 T cell activation is not yet understood ^22^ but evidence suggests that multiple CDRs of both the TCR-γ and -δ chains are involved as evidenced by site-directed mutagenesis ^23^ and demonstration of interdependence of CDR3s from both chains in PAg-reactivity ^24^.

*BTN3A* genes emerged with placental mammals but became defunct in many species, including mice and rats, similar to the co-evolving homologs of human Vγ9 (*TRGV9*) and Vδ2 (*TRDV2*) TCR genes ^25^. The human *BTN3A* gene family comprises *BTN3A1, BTN3A2*, and *BTN3A3* and was generated by gene duplication events during primate evolution ^26,27^. The gene products are expressed by most cell types including αβ and γδ T cells. The PAg-binding site of BTN3A1 is a highly conserved positively charged pocket formed by 6 amino acids of the intracellular B30.2 domain ^18,28^. Upon PAg-binding, this domain and the adjacent JM region undergo conformational changes ^19,29–31^ mandatory for mediating PAg-induced activation of Vγ9δ2 T cells.

Since their emergence in primates, BTN3A family members have diversified structurally and most likely functionally. Relative to BTN3A1, BTN3A2 lacks the entire B30.2 domain and parts of the JM region, while BTN3A3 bears an H381R substitution which abrogates PAg-binding to the pocket (numbering of amino acids as in Supplementary Fig. 1a) ^18^. The amino acid sequence identity of C-domains of the human BTN3As is about 90%, while the V domains of BTN3A1 and BTN3A2 are identical and that of BTN3A3 differs by a single conservative substitution (K66R) (Supplementary Fig. 1a) ^22^.

The contribution of BTN3A2 and BTN3A3 to PAg-mediated activation has been reported based on BTN3A family member knockdown studies in HeLa cells ^32^ and BTN3A knockout of 293T cells and various other cell lines ^33–35^; consistent with this, we have observed superior PAg responses when BTN3A1 was re-expressed in BTN3A1KO (*BTN3A1* gene inactivated) cells than in BTN3KO cells in which all three *BTN3A* genes are inactivated ^34^, suggesting that BTN3A1 needs the support of other BTN3A members. Moreover, association between BTN3A1 and BTN3A2, which occurs via their membrane-proximal IgC-like domains, was previously analysed, and retention motif-dependent ER sequestration of BTN3A1 was shown to be rescued by coexpression of BTN3A1 with BTN3A2 and resulting BTN3A1–3A2 heteromer formation ^33^. Nevertheless, how this relates to increased or altered PAg sensing functionality remains unclear. Furthermore, the exchange of the JM of BTN3A1 for that of BTN3A3 increases this activation ^36^. Nevertheless, how the BTN3A3JM contributes for enhanced function remains unknown.

In order to define minimal requirements of the different BTN3A molecules for PAg-induced activation of Vγ9Vδ2 T cells, we expressed combinations of wild-type and mutated BTN3A molecules in BTN3A-deficient 293T (BTN3KO) cells and demonstrated that the functional features of various BTN3A molecules can be merged in “super-BTN3” molecule, similar to a hypothesized primordial BTN3A present in species that encode single BTN3A isoforms such as Alpaca ^28,34,37^. We describe the BTN3A molecules as complexes in which for optimal function a division of labor takes place, whereby PAg-sensing is initiated by the B30.2 domain of one BTN3A chain and requires an intact IgV domain present within the paired BTN3A chain of each dimer. Our results show that the BTN3 JM region controls both trafficking and conformation of homomeric and heteromeric BTN3A complexes. In these complexes, the PAg-bound state is accompanied by binding of the BTN3A1-B30.2-PAg complex to the B30.2 domain of BTN2A1. These results not only clarify the molecular mechanism underlying PAg-mediated activation of Vγ9Vδ2 T cells but also have implications for γδ T cell activation by butyrophilin-related molecules such as BTNL or SKINT family members ^38^.

## Results

### Loss of function of VΔ3A1 compensated in heteromeric BTN3A complexes

**Figure 1:**
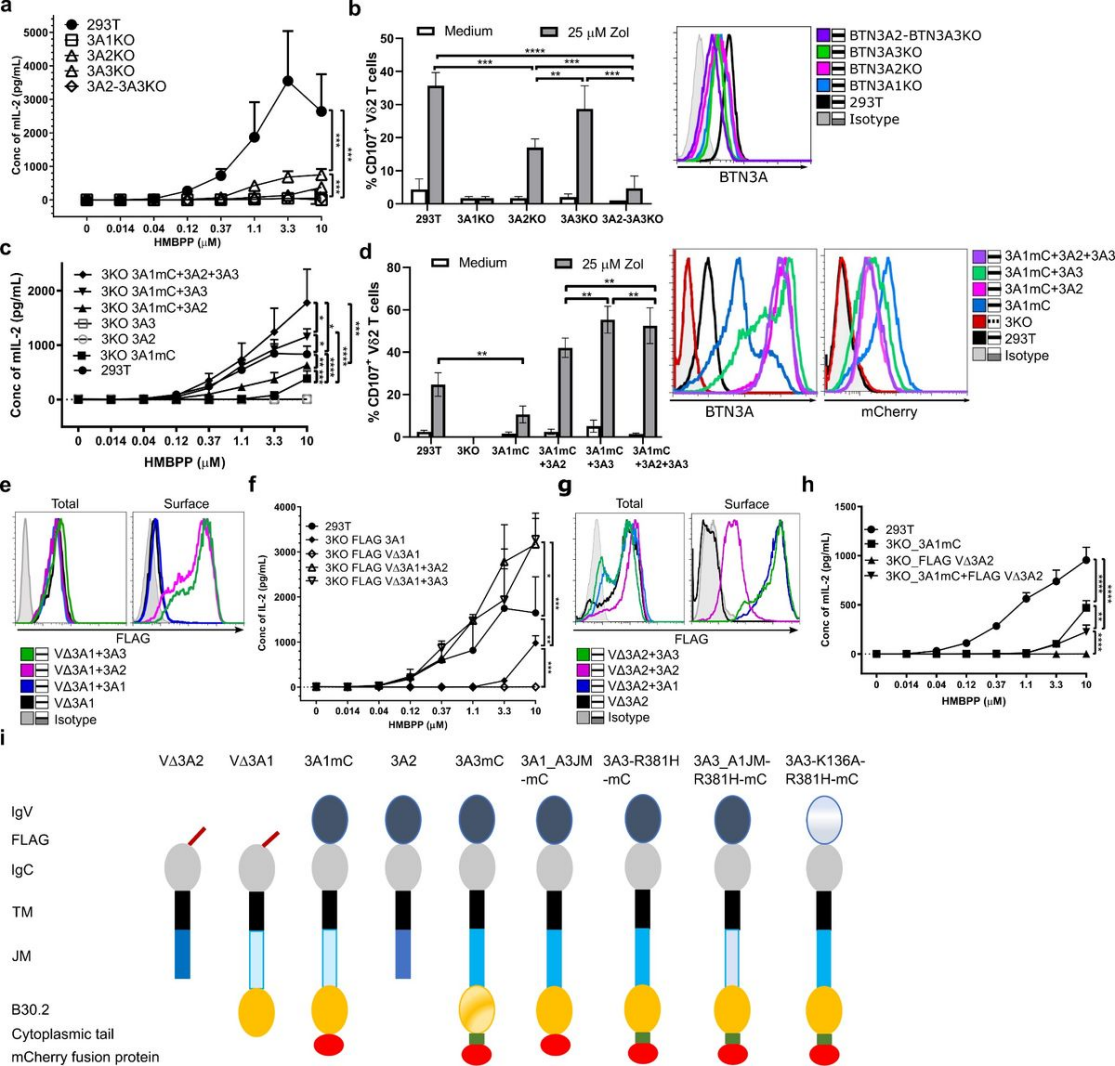
Loss of function of BTN3A1-V domain deleted molecules can be compensated in complexes with BTN3A2 or BTN3A3 molecules. **a** 293T and BTN3 isoform-specific knock-out cell lines were cocultured with titrated concentration of HMBPP and 53/4 human Vγ9Vδ2 TCR reporter cells. The activation of reporter cells was measured by mouse IL-2 ELISA (n-3). **b** 293T and BTN3 isoform-specific knock-out cell lines were pulsed with zoledronate and cocultured with HMBPP expanded primary Vγ9Vδ2T cells. The T cell activation was measured by immuno flow cytometry with CD107a expression as readout detected by anti-CD107a-PE and anti-Vδ2-FITC (n-3). Surface-expressed BTN3A of the above-mentioned cells detected by mAb 103.2 followed by anti-mouse F(ab’)2-APC conjugate (right). **c** 293T, BTN3KO (3KO) cells and 3A-transductants of 3KO were cultured and tested as in ***a*** (n-3). Not shown are the results of 293T 3KO as they are consistently non-stimulatory ^34^. **d** Above-mentioned presenting cells were tested as in *B*. Surface-expressed 3A-molecules of the above-mentioned cells detected by mAb 103.2 followed by anti-mouse F(ab’)2-APC conjugate and their corresponding total mCherry expression were presented as histograms (right). **e** Histograms representing the total and surface-expressed FLAG protein of fix-permeabilized and live 3KO cells transduced with FLAG-tagged IgVdeleted-BTN3A1 (VΔ3A1) alone or cotransduced with other 3A-molecules detected by anti-FLAG and anti-mouse F(ab’)2-APC conjugate were analyzed by FACS. **f** 3KO cells transduced with 3A2 or 3A3 and the cells from ***e*** were cocultured with 53/4 Vγ9Vδ2 TCR reporter and titrated concentration of HMBPP. The activation of reporter cells was measured by mouse IL-2 ELISA (n-3). **g** 3KO cells expressing FLAG-IgVdeleted-BTN3A2 (VΔ3A2) alone or together with other BTN3As were analyzed as in ***e***. **h** 293T wt and 3KO cells transduced with 3A1 and/or VΔ3A2 were analyzed as in G (n-3). **i** Schematic representations of different tagged constructs of 3A, 3A mutants, truncated 3A, and JM chimeras. The number of independent experiments was represented as n. Statistical significance in P-value is presented by asterisks (**** <0.0001; *** <0.001; ** <0.01; * <0.05; ns>0.05), and mean values with the SD are presented in graphs.

At first, we validated the necessity of all three isoforms for an optimal PAg response by testing inactivation of different BTN3A genes in 293T cells (– Fig. 1a – d) ^33,34^.To this end, we employed the murine reporter TCR-transductant MOP 53/4 r/mCD28 cell line (TCR-MOP), which shows no cross or self-presentation as is observed for human γδ T cells ^15,24,39^. The stimulation of the reporter TCR transductants is abrogated by BTN3A1 deficiency alone, or by knockout of both BTN3A2 and BTN3A3, and strong reduction of stimulation was observed for BTN3A2- than for BTN3A3-deficiency. A similar outcome was observed with primary human Vγ9Vδ2 T cells as responders, except that the loss of BTN3A3 alone was not as impactful as seen with TCR transductants. We also demonstrated the cooperation of BTN3A isoforms by transduction with 3A1 alone or in combination with 3A2, 3A3 or 3A2 plus 3A3 in 293T cells with all three BTN3A genes inactivated (BTN3KO cell line or 3KO). Additionally, 3KO cells that expressed 3A2 or 3A3 in the absence of 3A1 did not result in activation. Subsequently, all the experiments were performed in the 293T BTN3KO (3KO) ^34^ background and recombinant BTN3A derivatives were designated as 3A. A schematic overview of the constructs used in the study is provided in Fig. 1i.

Binding of BTN3A-V to the Vγ9Vδ2 TCR has been claimed ^40^ but could not be confirmed by surface plasmon resonance ^18^, isothermal titration calorimetry ^18^ or by staining of BTN3A1 transductants with Vγ9Vδ2 TCR-tetramers ^13^. To test the function of the human BTN3A family member V-domains, we generated recombinant BTN3A V-domain deletion mutants (VΔ) in which V domains were replaced by a FLAG-sequence preceded by a BTN3A1 leader sequence. If not explicitly stated, 293T BTN3KO cells (3KO) ^34^ were used as recipients for gene transduction. VΔ3A1 or VΔ3A2 were transduced alone or together with 3A1, 3A1mC, 3A2 or 3A3 (a schematic overview of the constructs is provided in Fig. 1i). VΔ3A3 was not tested since expression in 3KO cells failed. A sequence alignment of BTN3A molecules with relevant domains and regions marked is shown in Supplementary Fig. 1a. The transductants were sorted for similar BTN3A expression with the V-specific 103.2 mAb (Supplementary Fig. 1d and e) and stained for total expression (intracellular + surface expression of permeabilized and fixed cells) and surface expression (live cells) of the FLAG tag (Fig. 1e and g). Flow cytometry revealed that the VΔ3A1 transductant displayed no surface staining of the FLAG-tag unless a heterologous 3A-molecule was co-expressed (3A2 or 3A3 but not 3A1), and this result was confirmed with confocal microscopy (Supplementary Fig. 1f). Cell surface FLAG-staining of VΔ3A2 also required co-transduction of intact 3A-molecules. In this case, the reconstitution of FLAG-epitope surface expression by homologous 3A2 was weak but efficient for the heterologous 3A1 and 3A3. In conclusion, lack of the V-domain disrupts the BTN3A trafficking to cell surface and staining of such VΔ-domain constructs (FLAG-VΔ3A) required co-expression of appropriate full-length BTN3A molecules.

Next, we tested for HMBPP-induced stimulation of the MOP TCR-transductant cell line ^15,24,39^. 3KO cells transduced with VΔ3A1 and 3A2, or VΔ3A1 and 3A3 stimulated better than wild-type 293T cells, while cells co-expressing VΔ3A2 and 3A1 stimulated even worse than cells expressing only 3A1 (Fig. 1f and h). This reduced efficacy was not an effect of the FLAG-tag (Supplementary Fig. 1c). Notably, protein domains contained in the complexes of VΔ3A1 and 3A2, or VΔ3A2 and 3A1, are identical (Fig. 1a and Fig. 3g), indicating that functional differences of the complexes result from the different localization of domains within the complexes, as will be discussed later.

### The Jm Region Regulates Btn3a-protein Interaction And Function

**Figure 2:**
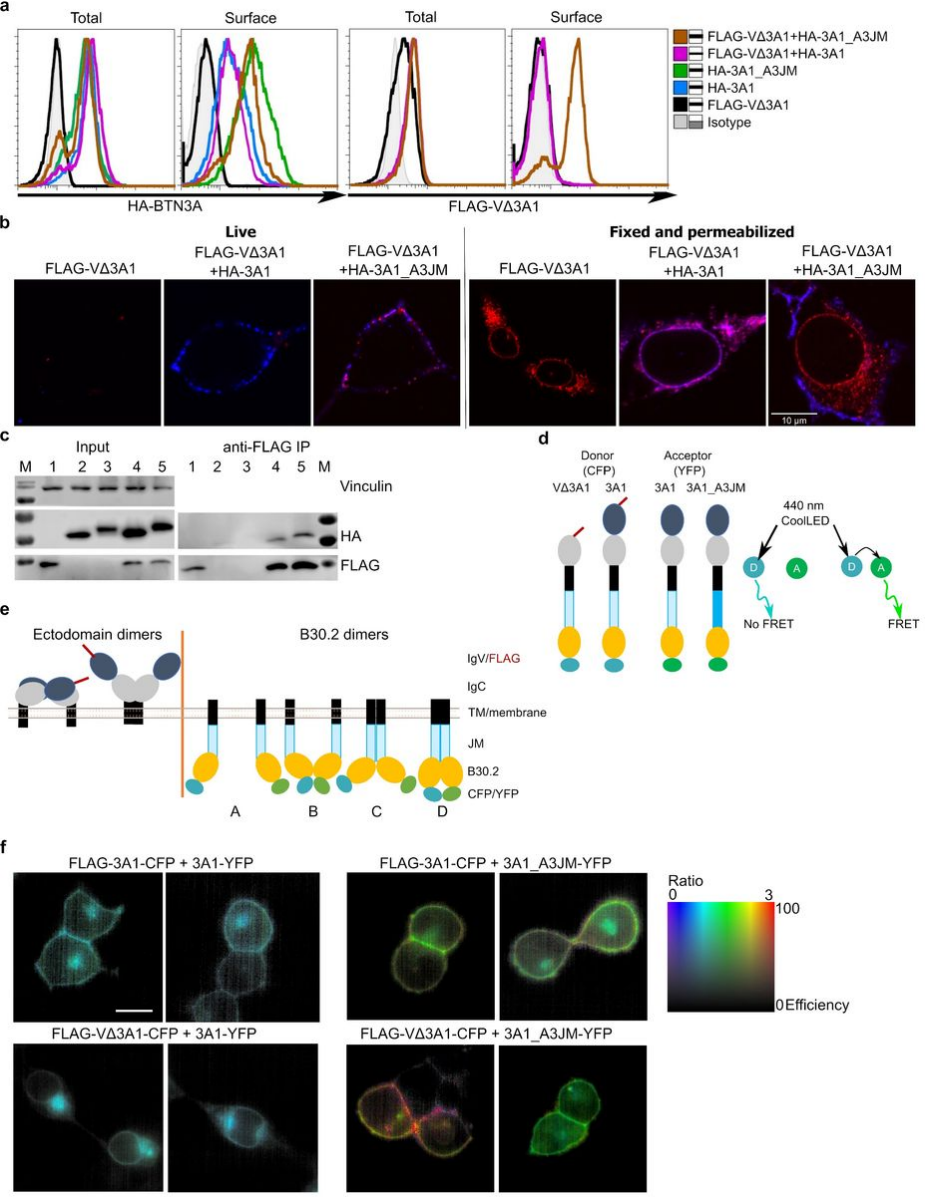
The JM region regulates BTN3A-protein and function. **a** 293T 3KO cells transduced with FLAG-VΔ3A1 alone and or cotransduced with N-terminus HA-tagged 3A-JM chimeras were analyzed in FACS for the total and surface expression of HA-3A molecules (Left) and FLAG-VΔ3A1 (right). The measurements were presented as histograms. **b** Live (left) and fix-permeabilized (right) 3KO cells transduced with FLAG VΔ3A1, cotransduced with HA-3A1 or HA-3A1_A3JM chimera were stained with mouse anti-FLAG and rabbit anti-HA followed by anti-mouse-Alexa Fluor 647 (red) and anti-rabbit Alexa Fluor 568 (blue), respectively. **c** 3KO cells transduced with FLAG-VΔ3A1, HA-3A1, HA-3A1_A3JM, FLAG-VΔ3A1 + HA-3A1, and FLAG-VΔ3A1 + HA-3A1_A3JM were labeled as 1 – 5, were subjected to anti-FLAG immunoprecipitation (IP) and samples were blotted against human vinculin (input, top), FLAG (middle) and HA (bottom) for their input (left) and immunoprecipitated proteins (right) (n-2).**d**Schematic presentation of FLAG-VΔ3A1-CFP, FLAG-3A1-CFP, 3A1-YFP and 3A1_Y3JM-YFP constructs (left), scheme describing the FRET with 440 LED laser, D is the donor (CFP), A is the acceptor (YFP) and A will emit a signal when exited by D if it is close proximity showing FRET. **e** Schematic presentation of probable ectodomain dimers and cytoplasmic B30.2 dimers based on the literature. Different cytoplasmic dimers expected were marked as A, B, C & D. **f** Ratiometric FRET analysis of 3KO transduced with 3A1-YFP and FLAG-3A1-CFP (upper left) or FLAG-VΔ3A1-CFP (lower left); 3KO transduced with 3A1_A3JM-YFP and FLAG-3A1-CFP (upper middle) or FLAG-VΔ3A1-CFP (lower middle); FRET ratio (FR) calculated chart (right).

A major difference when comparing BTN3A1 relative to both BTN3A2 and BTN3A3 is their JM region (Supplementary Fig. 1a). To address its role in BTN3A isoform interaction and function, FLAG-VΔ3A1 was coexpressed with HA-tagged 3A1 or 3A1 containing the JM of 3A3 (3A1_A3JM). In cells with similar total levels (intracellular and cell surface) of FLAG-VΔ3A1, its surface expression was detected by flow cytometry only when co-transduced with 3A1_A3JM but not native 3A1 (Fig. 2a). This finding suggests that the BTN3A1 JM region might hinder formation of fully functional BTN3A complexes while the heterologous BTN3A3 JM region may support such complexes. The ratio of cell surface to total expression was also considerably higher for HA-3A1_A3JM compared to wild-type HA-3A1 (Fig. 2a). This demonstrates the capacity of 3A3JM to alter the pattern of cellular distribution of 3A1_A3JM as well as the associated FLAG-VΔ3A1. Similar observations were made using confocal microscopic examination of immuno-stained live 3KO cells expressing FLAG-VΔ3A1 and HA-3A1 or HA-3A1_A3JM (Fig. 2b). Immuno-staining with anti-FLAG antibody detected the FLAG-VΔ3A1 (red) at the cell surface under live conditions only when co-transduced with HA-3A1_A3JM (right) but not with HA-3A1 (center). Furthermore, HA-3A1 or HA-3A1_A3JM (blue) proteins were clearly detected at the cell surface by anti-HA antibody, validating the presence of full-length proteins at the cell surface. Under fixed-permeabilized conditions (right hand panels) FLAG-VΔ3A1 was detectable at the cell surface only if colocalizing with HA-3A1_A3JM (violet, right). In contrast to live conditions, clear colocalization of FLAG-VΔ3A1 and HA-3A1 was observed in cytoplasmic vesicles. Notably, when FLAG-VΔ3A1 was coexpressed with HA-3A1_A3JM, HA-tag was detected largely at the membrane, with hardly any detectable in cytoplasmic vesicles. Similar observations were made with FLAG-VΔ3A1-CFP coexpressed with 3A1-YFP or 3A1_A3JM-YFP (Supplementary Fig. 2b). Finally, microscopic examination of these cells revealed the altered trafficking of 3A1_A3JM attributed to 3A3JM.

We performed immunoprecipitations (IP) using the cells mentioned above to extend our findings to biochemical interactions. Cell lysates were subjected to anti-FLAG IP and subsequent anti-HA western blot (Fig. 2c). In line with the colocalization of FLAG-VΔ3A1 with HA-3A1 under fixed-permeabilized conditions and at the cell surface for FLAG-VΔ3A1 with HA-3A1_3A3M, IP demonstrated potential interactions between FLAG-VΔ3A1 with HA-3A1 or HA-3A1_A3JM but did not show any differences in the quantities of co-precipitated HA-proteins. The differential size of HA-3A1_A3JM and HA-3A1 in the immunoblot coincided with their differential localization and trafficking.

### Btn3a3 Jm Promotes Close Association Of B30.2 Domains In Btn3a Complex

Although VΔ3A1 association was observed with both HA-3A1 and HA-3A1_A3JM constructs in IP, the differential surface expression of VΔ3A1 led us to postulate that the resulting heteromeric 3A complexes adopted different conformations. FRET analysis was used to test the interaction between fluorescent fusion proteins and to infer the conformation or mode of association between 3A-molecules within homomers or heteromers. For FRET assays, 3KO co-transductants of FLAG-VΔ3A1-CFP or FLAG-3A1-CFP and 3A1-YFP or 3A1_A3JM-YFP were generated (Fig. 2d). FRET ratio was measured as stipulated in the methods section and acquired images are presented as ratiometric images (Fig. 2f).

The setup was optimized with 3KO single transductants of FLAG-3A1-CFP, and 3A1-YFP/3A1_A3JM-YFP constructs; the intensity 480/30 and 535/40 filters were similar with CFP constructs, and no image was visualized with YFP constructs as YFP was not excited by a 440nM CoolLED (Supplementary Fig. 2c).

The full-length 3A1-CFP/VΔ3A1-CFP coexpressed with 3A1-YFP displayed no FRET (Fig. 2f, left panel) and yielded images with similar intensities with both the filters, suggesting no interaction between CFP and YFP either on the cell membrane or in the cytoplasmic compartments (Supplementary Fig. 2c). However, 3A1-CFP co-expressed with 3A1_A3JM-YFP revealed high FRET predominantly at the membrane (Fig. 2f, upper right), and with the increased intensity with the 530nM-filter (Supplementary Fig. 2c).

Even stronger FRET was observed at the membrane when FLAG-VΔ3A1-CFP was co-expressed with 3A1_A3JM-YFP (Fig. 2f, lower right). This was consistent with observations from immune staining and confocal microscopy (Fig. 2b and Supplementary Fig. 2b), where 3A1_A3JM was overwhelmingly detected at the cell membrane but not in cytoplasmic organelles, and in spite of the predominant cellular retention of the VΔ3A1 protein, detectable levels of FLAG-tagged protein managed to reach the cell membrane when cotransduced with 3A1_A3JM.

Collectively, these data suggest that expression of FLAG-VΔ3A1-CFP or 3A1-CFP with 3A1-YFP led to3A-complexes where B30.2 domains are distantly spaced. On the contrary, co-expression of FLAG-VΔ3A1-CFP or 3A1-CFP with 3A1_A3JM suggests the formation of heteromers in which their respective B30.2 domains are in FRET-able distance as predicted (Fig. 2e). We hypothesized that an equivalent type of association occurs for the intracellular domains of VΔ3A1 or 3A1 when co-expressed with 3A2 but could not address this using the same methodology due to the different lengths of the intracellular domains and consequently of the adjacent fluorophores, which would confound FRET efficiency.

### A division of labor in BTN3A heteromers and super-BTN3 homomers

**Figure 3:**
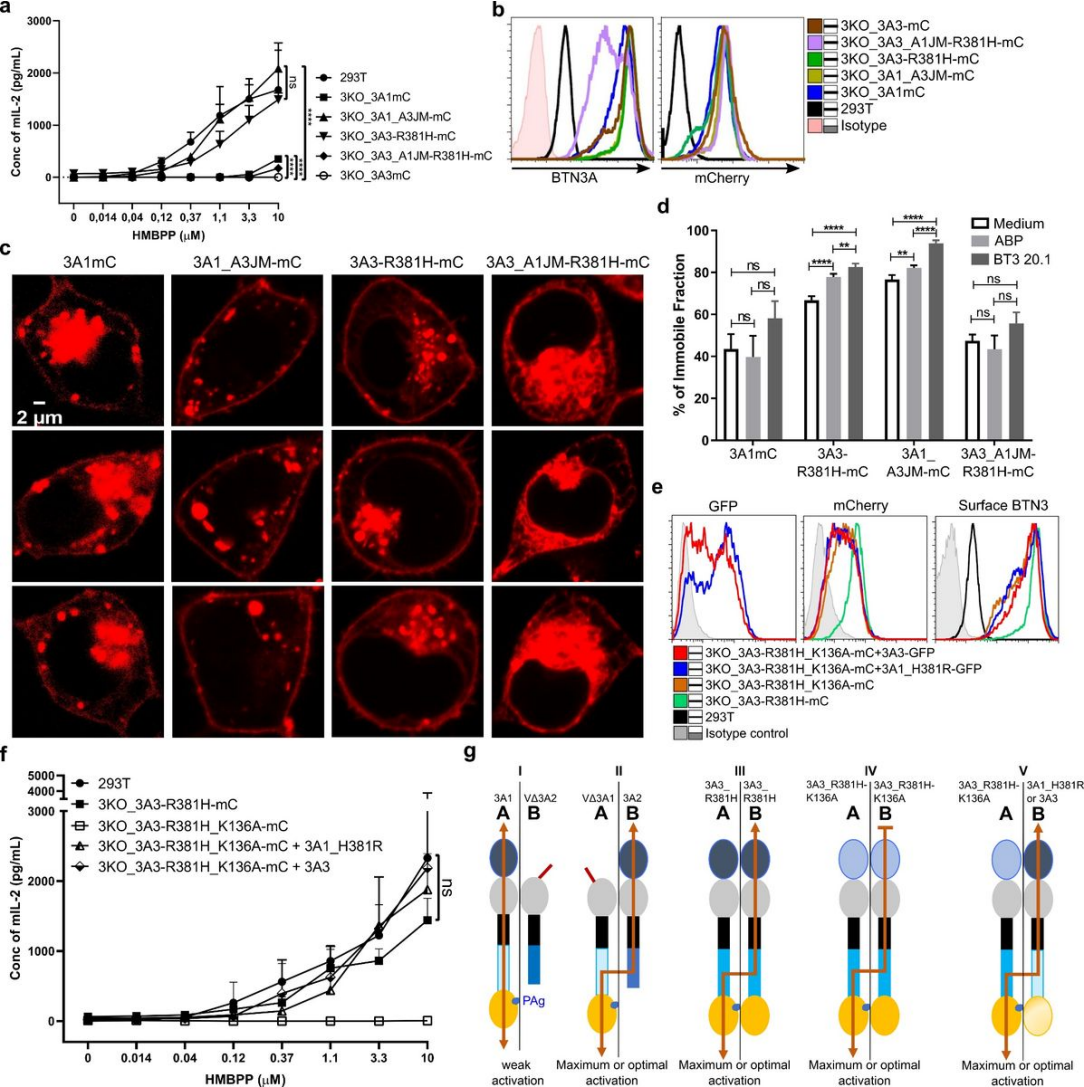
Homomeric 3A3_JM and Heteromeric 3A_JM promote optimal stimulation via inter-BTN3 PAg signaling. **a** 293T and 3KO transductants of 3A1, 3A3, 3A3_R381H, or 3A_JM chimeric constructs were cultured and tested as in A (n-3). **b** Surface-expressed 3A-proteins of the above-mentioned cells detected by mAb 103.2 followed by anti-mouse F(ab’)2-APC conjugate (left) and their corresponding total mCherry expression (right) were presented as histograms. **c** The cellular distribution of BTN3A-mC fusion constructs is presented as images captured by confocal microscopy. **d** mCherry fusion constructs of 3A or 3A-JM chimera transduced 3KO cells were subjected to FRAP and the percentage of the immobile fraction of BTN3A-mC was measured. The number of cells (n) subjected to FRAP for 3KO_3A1mC (n-15), and other cell types (n-10) for each condition. **e**293T, 3KO transduced with mCherry fusion constructs of 3A3_R381H, 3A3_K136A_R381H, and cotransduced with eGFP reporter constructs of 3A1_H381R or 3A3 were analyzed by FACs for their total mCherry, total GFP, and surface-expressed BTN3As detected by mAb 103.2 and anti-mouse F(ab’)2-APC conjugate, the measurements were presented as histograms (bottom right). **f**The above-mentioned cells were tested as in ***a*** (n-3). The predicted intermolecular signaling within the BTN3A proteins viz 3A3_R381H, 3A3-K136A-R381H, and 3A3/3A1_H381R and the observed stimulation strength was presented as a scheme in **g** III, IV and V, respectively. **g** Schematic presentation of predicted intermolecular signaling within the BTN3A proteins correlated to the observed outcomes in terms of 53/4 human Vγ9Vδ2 TCR reporter activation strength with antigen-presenting cells (3KO) expressing VΔ3A2 and 3A1 (I), VΔ3A1 and 3A2 (II) including the 3A-constructs mentioned in **f**. Statistical significance in P-value is presented by asterisks (**** <0.0001; *** <0.001; ** <0.01; * <0.05; ns>0.05), and mean values with the SD were presented in graphs.

Alpaca-like species demonstrating single BTN3-dependent PAg responses led us to postulate a single BTN3 molecule as a primordial requirement, and it was of interest to generate such a BTN3 protein, which encompasses requisite domains for the PAg-dependent response. To this end, 3KO cells transduced with mCherry (mC) fused to 3A1 (3KO_3A1mC), 3A3 gain of function mutant R381H (3KO_3A3-R381H-mC), 3A1 with the JM of BTN3A3 (3KO_3A1_A3JM-mC) and finally with a gain of function 3A3 mutant possessing JM of 3A1 (3A3_A1JM_R381H-mC) were analyzed (Fig. 3a–d). In the functional assay (Fig. 2a), cells expressing a 3A-proteins with a functional PAg sensing B30.2 domain and the 3A3 JM region were indistinguishable from 293T cells, whereas cells co-expressing 3A1-mC and 3A3_A1JM_R381H-mC that possess the 3A1 JM region were very poor stimulators, and as expected 3A3 expressing cells did not stimulate at all. Analysis of recombinant BTN3A protein distribution in these cells revealed that despite a similar degree of mCherry fusion protein expression (Fig. 3b) the cells exhibited pronounced differences in intracellular localization and in the formation of mCherry aggregates (Fig. 3c). In all cases, cells expressing 3A-molecules bearing exclusively 3A1JM displayed a higher degree of intracellular retention of fluorescent complexes than their 3A3JM expressing counterparts, which displayed enhanced expression at the plasma membrane (Fig. 3c). Finally, we tested the effects of the aminobisphosphonate pamidronate, and the agonistic mAb 20.1, on the cell surface immobility of 3A-molecules by FRAP (Fluorescence Recovery after Photobleaching) ^15^. Constructs with a 3A1JM displayed no increased immobilization whereas those with a 3A3JM did (Fig. 3d). Notably, medium controls of the cells expressing the 3A3JM-containing constructs also displayed a higher degree of immobilization than that of the transductants with 3A1JM-containing constructs (3A1mC and 3A3-A1JM-R381H-mC), which is consistent with the reported higher background stimulation for activation of short term Vγ9Vδ2 T cell lines by 293T transfected with 3A1_A3JM ^36^ or 3A3_A1_B30.2 and 3A3_R381H ^18^. Likewise, cells expressing 3A1-mC plus 3A2–3A3 (Supplementary Fig. 3a) behaved analogously to cells expressing the 3A3 JM-containing constructs in terms of intracellular trafficking and aggregate formation. Furthermore, native gel electrophoresis of solubilized membrane extracts revealed very large 3A1-mC complexes when prepared with detergent Brij 96 and Triton X100 (Supplementary Fig. 3b). In contrast, membranes solubilized with digitonin, which binds to cholesterol, massively reduced the size of 3A1mC molecular complexes. In the presence of 3A2 and 3A3 these complexes were dissociated into two complexes of less than 440 kDa apparent MW ^18^. Altogether the 3A3-JM-containing constructs can substitute for “help” for 3A1 JM by 3A2 or 3A3 in terms of stimulation capacity, cellular trafficking of 3A proteins, and formation of molecular clusters.

So far, we showed that functional impairment of 3A-heteromer formation coincides with reduced stimulatory capacity. Surprisingly, VΔ3A1 + 3A2 and VΔ3A2 + 3A1 complexes stimulated quite differently, although the surface expression of each heteromer was similar (Fig. 1f–h). Moreover, as depicted in Fig. 3g, both complexes possess sequence-identical protein domains and differ only in the relative arrangement of the V domains. In one case the IgV domain is located on the PAg-binding protein (3A1), in the other on the pairing chain (3A2). This feature relates back to a previous report on V-domain mutants (K136A) affecting PAg-mediated stimulation ^41^ where heteromers of 3A2_K136A and 3A1 lost stimulatory potential while heteromers of 3A1_K136A and 3A2 did not. To test whether similar effects were also observed for a homomeric “super” BTN3A” (3A3_R381H), a mutant with a substitution at position 136 was generated (3A3_R381H_K136A-mC). 3A3_R381H_K136A-mC was co-expressed with one of two different PAg-binding-insufficient BTN3A-IRES-GFP reporter constructs (3A3 (GFP) or 3A1_H381R(GFP)) (Fig. 3e). Stimulation was successfully detected with both the co-transductants where the PAg-binding site and wild type V-domain were located on different molecules (Fig. 3f–g), which is consistent with the differential stimulatory capacity of VΔ3A1 + 3A2 vs VΔ3A2 + 3A1 transduced cells. Altogether, these results suggest PAg-binds to one BTN3A molecule that via the JM region is connected to a paired BTN3A molecule whose intact V-domain is essential for PAg sensing mediated via the Vγ9Vδ2 TCR.

### A Structural Rationale For Heteromeric Btn3a Coiled-coil Assembly

**Figure 4:**
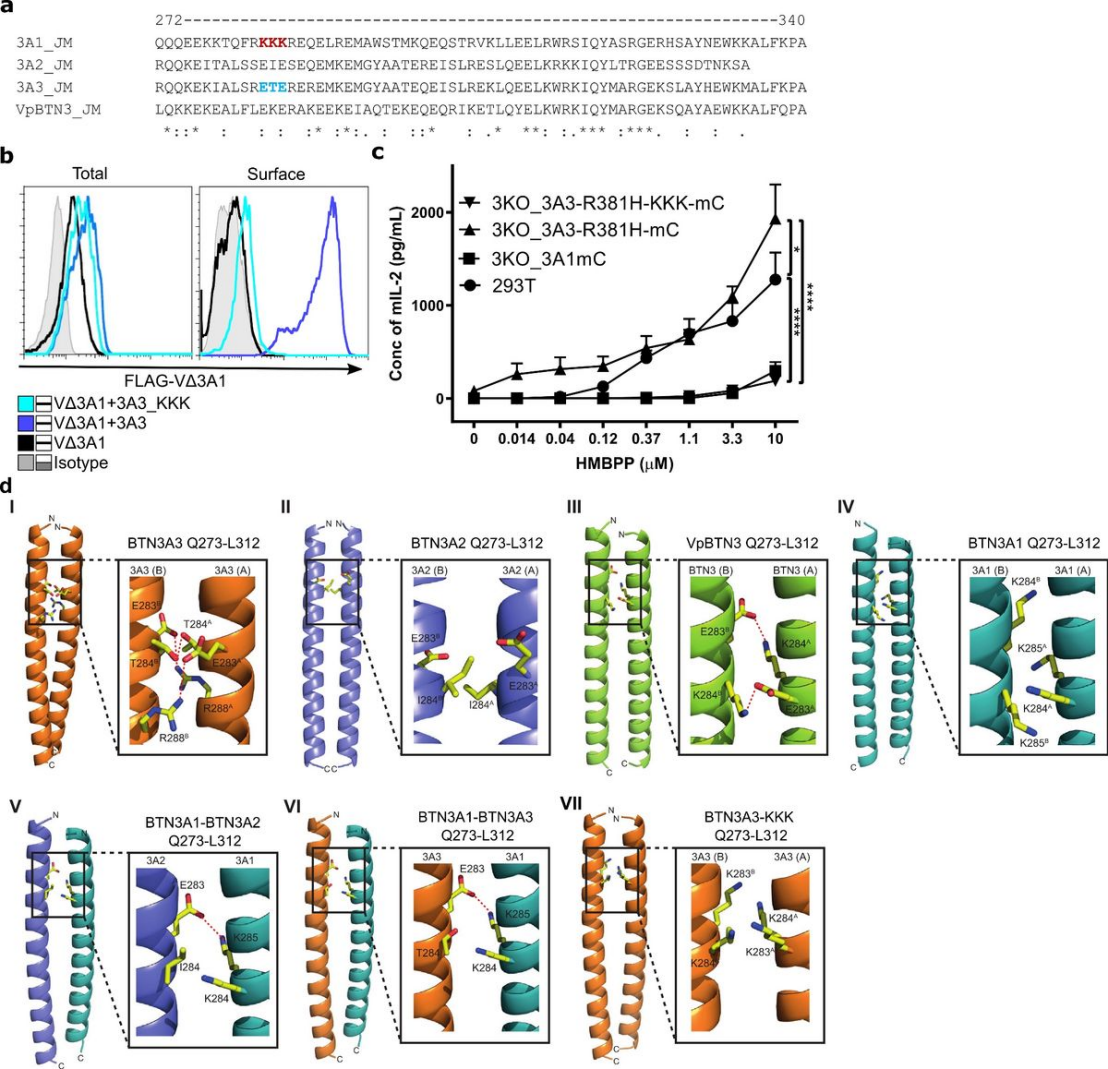
JM regions modulate the conformation of BTN3A dimers. **a** Amino acids encoding juxtamembrane (JM) region of BTN3A1, BTN3A2, BTN3A3, and alpaca BTN3 (Vp) were aligned, and KKK and ETE residues of BTN3A1 and BTN3A3 were marked in red and blue, respectively. **b** Total and surface-expressed FLAG protein of permeabilized and live 3KO cells transduced with FLAG VΔ3A1 alone or cotransduced with 3A3 or 3A3_KKK mutant detected by anti-FLAG and anti-mouse F(ab’)2-APC conjugate were shown as histograms. **c** 3KO cells transduced with 3A1mC, 3A3_R381H-mC, or 3A3_R381H_KKK-mC mutant were cocultured with 53/4 Vγ9Vδ2 TCR reporter cells and titrated concentration of HMBPP. The activation of reporter cells was measured by mouse IL-2 ELISA (n-3). **d** Models of the BTN3-JM coiled-coil dimers. Models of the predicted JM coiled-coil dimers Q273–L312 were generated using CCBuilder2 (see Methods). Dimer interface residues at positions 283–285 are shown as ball and stick. I) BTN3A3 coiled-coil homodimer, II) BTN3A2 coiled-coil homodimer, II) Alpaca BTN3 (VpBTN3) coiled-coil homodimer, IV) BTN3A1 coiled-coil homodimer, V) BTN3A1-BTN3A2 coiled-coil heterodimer, VI) BTN3A1-BTN3A3 coiled-coil heterodimer, VII) BTN3A3-KKK (replacing ETE with KKK at positions 283–285) coiled-coil homodimer. Polar interactions are highlighted (red dashed lines). Each monomer within the homodimer has been labeled A or B. Statistical significance in P-value is presented by asterisks (**** <0.0001; *** <0.001; ** <0.01; * <0.05; ns>0.05), and mean values with the SD were presented in graphs.

To probe the differential impact of the JM region on BTN3A function, we compared the sequence of BTN3A1JM to that of other BTN3A molecules (Supplementary Fig. 1a and Fig. 4a). We noted that the JM of BTN3A1 contains a positively charged lysine-triplet (KKK) (position 283–285) while BTN3A2, BTN3A3, and alpaca BTN3A possess two negatively charged glutamic acid residues (ExE) at this position (Fig. 4a). Moreover, the substitution of the BTN3A3 ETE motif by KKK (3A3-KKK) abolished the rescue of surface expression of FLAG-VΔ3A1 and reduced the stimulatory activity to that of 3A3-R381H-KKK-mC (Fig. 4b and c). This suggested that this triplet motif is essential for the JM-mediated interaction of 3A1 and 3A3 molecules. Interestingly, replacement of KKK of BTN3A1 by ETE (3A1_ETE) did not rescue FLAG-VΔ3A1 cell surface expression and did not change the stimulatory capacity of the 3A1, suggesting other regions of the JM may also be involved in controlling cooperation and trafficking of associated 3A-molecules (Supplementary Fig. 4a and b).

To probe the molecular basis of these effects, we carried out molecular modeling of the coiled-coil region of the BTN3A isoforms. We restricted these efforts to the 273–312 region that was previously strongly predicted to form a coiled-coil domain by mediating BTN3A dimer interactions ^42^, within which the BTN3A1 KKK ‘triplet region’ is located (283–285), and employed a parametric α-helical coiled coil prediction methodology (CCBuilder 2.0) ^43^.

These efforts first highlighted the potential of human BTN3A1, BTN3A2, BTN3A3, and also the single alpaca isoform VpBTN3, to each form biophysically plausible homodimers via intermolecular coiled-coil interactions, stabilized in each case by numerous polar and non-polar interactions at the inter-helical molecular interface. Of note, these models predicted inter-helical interactions mediated by the 283–285 triplet residues that could partly account for differential stability and conformation (Fig. 4d), and therefore surface expression and functionality (Fig. 4b–c). In the BTN3A3 homodimer, E283 and T284 were predicted to form stabilizing hydrogen-bonding interactions to equivalent residues of the opposing helix, with the involvement of R288 from each monomer; in contrast E285 was solvent exposed and not involved in interhelical contacts (Fig. 4d I). In BTN3A2, I284 was the sole mediator of interhelical triplet region interactions comprised of non-polar interface contacts with the corresponding residue of the opposing helix (Fig. 4d II); unlike BTN3A3, E283 and E285 were solvent exposed and uninvolved in intermolecular contacts. While biophysically feasible, the relative stability of this arrangement was unclear. Nevertheless, it is consistent with the weaker surface expression of VΔ3A2 when coexpressed with 3A2 compared to that of coexpression with 3A1 and 3A3. Similar to human BTN3A3, modelling of the single alpaca-encoded ‘superagonist’ isoform, VpBTN3, indicated involvement at the inter-helical interface of E283 and K284, which mediated reciprocal salt bridge interactions with the same pair of residues from the opposing monomer (Fig. 4d III). Notably, for the BTN3A1 model the indicated ‘KKK’ at 283–285 region was arranged differently, with 284 and 285 positioned at the inter-helical interface and 283 solvent exposed and uninvolved (Fig. 4d IV). Most importantly, this model predicted the positively charged K284 and K285 were directly facing the same residues from the opposing monomer at the interface (Fig. 4d IV). This arrangement is likely to be energetically highly unfavorable and destabilize the BTN3A1 homodimer via electrostatic repulsion; moreover, consistent with results from FRET analyses (Fig. 2), it may favor a weaker inter-molecular association. Therefore, while biophysically feasible, BTN3A1 modeling highlights the KKK motif of BTN3A1 is likely to disfavor homodimer formation in a way that is not predicted to occur with other isoforms.

Modelling approaches also shed light on heteromeric interactions. BTN3A1/3A2 (Fig. 4d V) and BTN3A1/3A3 (Fig. 4d VI) coiled-coil models highlighted not only a loss of the interhelical electrostatic repulsion evident from the 283–285 region of BTN3A1 homodimers (Fig. 4d IV), but also predicted a favorable salt-bridge interaction from K285 of 3A1 to E283 of BTN3A2/3A3. This was consistent with more stable coiled-coil heterodimers relative to the BTN3A1 homodimer, including a potential for closer intermolecular association between the two BTN3A chains in this context, consistent with the results of the FRET analyses. Of note, modelling of BTN3A3 mutated to incorporate the KKK motif of BTN3A1 at 283–285 (Fig. 4d VII) indicated that close opposition of K283 and K284 to identical residues across the inter-helical interface. Although this differed from the predicted native BTN3A1 dimer interface, where K284 and K285 are localized to the dimer interface, it was nevertheless likely to substantially destabilize the BTN3A3-KKK dimer and was entirely consistent with the pronounced deleterious effect of the BTN3A1 JM region (Figs. 1 and 3) and KKK motif (Fig. 4) on both surface expression, conformation, and functionality.

Finally, inspection of the models strongly indicated extra-triplet effects contribute to differential homodimer and heterodimer stability (Supplementary Fig. 4, Supplementary Material). In particular, the 276–278 region appeared particularly significant (Supplementary Fig. 4c I-VI), as it was predicted to form stabilizing non-polar (BTN3A2 homodimers) (Supplementary Fig. 4c II), or salt bridge interactions (BTN3A3 homodimer, alpaca BTN3 homodimer, BTN3A1/A2 heterodimer, BTN3A1/A3 heterodimer) (Supplementary Fig. 4c III-VI), whereas in BTN3A1 the presence of K277 and K278 introduced electrostatic repulsion at the dimer interface (Supplementary Fig. 4c I). Moreover, the intermolecular packing interactions mediated by L280 in all other isoforms were lost in BTN3A1 homodimers (Supplementary Fig. 4c VII-X), in which the polar residue (Q) at this position was predicted to be solvent-exposed (Supplementary Fig. 4c VII). In summary, interhelical interactions outside of the 283–285 region clearly also preferentially destabilize BTN3A1 homomers relative to both BTN3A2/3 homomers, and also relative to heteromers involving BTN3A1 and BTN3A2/A3. This provides a molecular explanation for the observation that introduction of the 283–285 ETE sequence of BTN3A3 into 3A1 is insufficient to confer substantially increased expression and functionality (Supplementary Fig. 4a-b).

### 4-M-HMBPP disrupts the interaction of BTN3A1-BTN2A1 B30.2 domains

**Figure 5:**
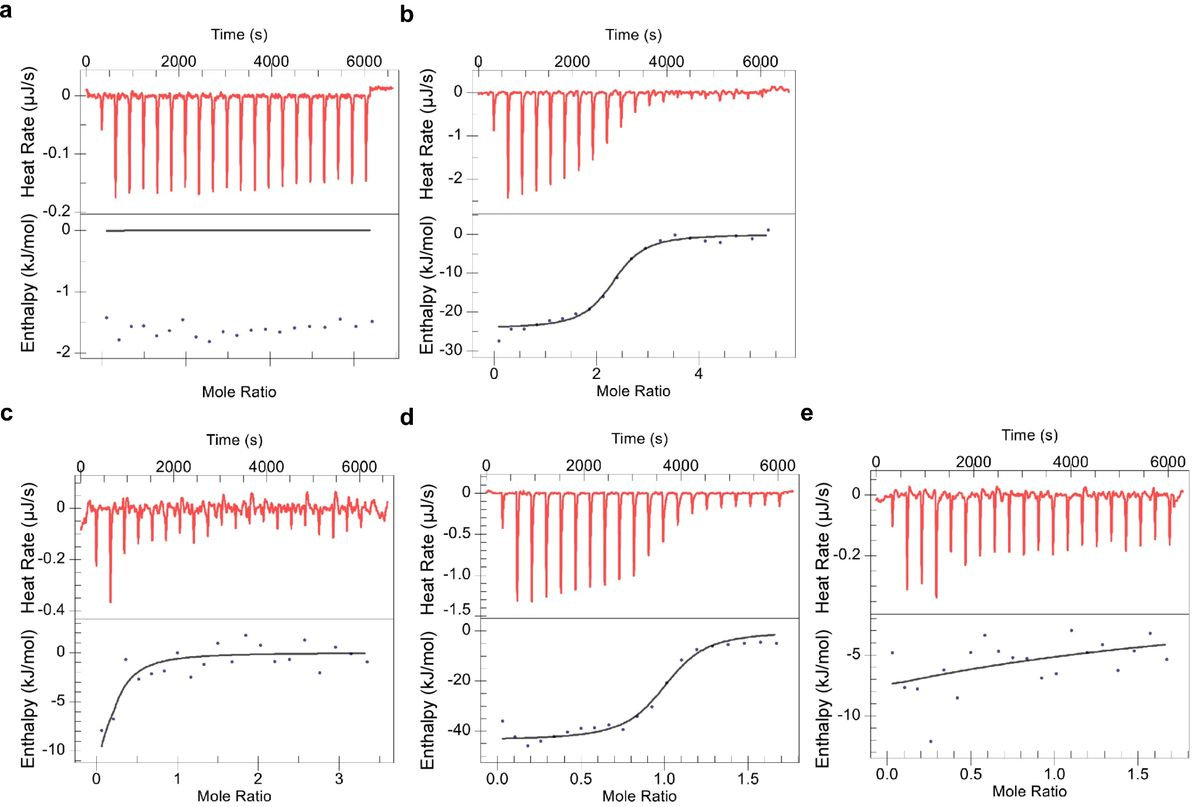
4-M-HMBPP bound BTN3A1 did not interact with the BTN2A1-B30.2 domain. ITC titrations show that 4-M-HMBPP binds to BTN3A1 but does not support the binding of BTN3A1 to BTN2A1. **a** Titration of 960 μM 4-M-HMBPP into the buffer. bTitration of 960 μM 4-M-HMBPP into 60 μM BTN3A1 BFI. **c** Titration of 600 μM BTN2A1 ID271 into 60 μM BTN3A1 BFI. **d** Titration of 300 μM BTN2A1 ID271 into a mixture of 60 μM BTN3A1 BFI and 120 μM HMBPP. **e** Titration of 300 μM BTN2A1 ID271 into a mixture of 60 μM BTN3A1 BFI and 120 μM 4-M-HMBPP. Results are representative of n-3 independent experiments.

We next compared HMBPP and 4-M-HMBPP, a HMBPP derivative incorporating a bulky head group that permits HMBPP-like binding to the BTN3A1-B30.2 domain with reduced stimulatory capacity that has been suggested to result from an “aberrant” BTN3A1-B30.2 homodimer ^44^. We previously demonstrated that the intracellular domains of BTN2A1 and BTN3A1 interact, but only in the presence of a potent PAg such as HMBPP ^20^. Here we examined the ability of 4-M-HMBPP to support this interaction. We confirmed a robust binding interaction between 4-M-HMBPP and the BTN3A1 full intracellular domain (BFI) (Fig. 5b), albeit with a somewhat lower binding affinity of 2.9 μM that may result from different 3A1 constructs or compound purities. Next, we titrated BTN2A1 intracellular domain (ID271) into 3A1 BFI. In agreement with our prior study, no interaction was observed in the absence of PAg (Fig. 5c) while in the presence of HMBPP, a strong interaction was observed (K_D_, 0.8 μM) (Fig. 5d) which coincides with the finding reported in a recent preprint by the Zhang group ^21^. However, in the presence of 4-M-HMBPP, no binding occurred between BTN2A1 ID271 and BTN3A1 BFI (Fig. 5e) as shown in Table 1. Therefore, we can conclude that while 4-M-HMBPP binds to BTN3A1, yet it does not allow it to engage subsequently with BTN2A1. Together, binding of PAg to BTN3A1 in the BTN3A heteromer allows it to interact with BTN2A1 homodimer to promote T cell activation.

## Discussion

This study addresses the contribution of BTN3A protein domains and their binding partners to PAg-induced Vγ9Vδ2 T cell activation. Firstly, it demonstrates a crucial role for the V-domain for cell surface expression of BTN3A molecules. Secondly, the impaired trafficking of BTN3 lacking its membrane distal IgV-domain could be rescued by partnering preferentially BTN3 molecules possessing the equivalent domain. Thirdly, the functional contribution of the BTN3A membrane distal IgV domain to PAg stimulation can be compensated by the paired BTN3A molecule. Such compensation of loss of function BTN3A1-V constructs by residual levels of BTN3A2/BTN3A3 isoforms could explain the observation that BTN3A1 V-domain mutants expressed in BTN3A-knockdown 293T cells did not display any phenotype ^43^. It may also explain why a human Vγ9Vδ2 TCR transductant (TCR-MOP) that does not react to HMBPP-pulsed 3KO cells transduced with an alpaca BTN3(V-C)-human intracellular domain chimera but gains responsiveness when the same construct was transduced into BTN3A1KO cells, suggesting that chimera comprising heteromers involving V domains of endogenous BTN3A2 and/or BTN3A3 may engage with the human TCR or permit its ligation by an associated ligand ^34,37^.

BTN3A2 as well as BTN3A3 reconstituted surface expression of VΔ3A1 and the resulting complexes permitted PAg-induced Vγ9Vδ2 TCR-mediated activation as efficiently as naturally occurring BTN3A heteromers or “super” BTN3As. In striking contrast simultaneous expression of VΔ3A2 with BTN3A1, despite rescuing VΔ3A2 cell surface expression, failed to increase BTN3A1 mediated stimulation. Since the protein domains of surface-expressed 3A1-VΔ3A2 complexes and of 3A2-VΔ3A1 are identical we conclude that localization of the V-domain within the complex is crucial for HMBPP-mediated stimulation. Such a topological effect could also explain the differential stimulation by 3KO cells co-expressing V-domain mutated BTN3A1 and wild-type BTN3A2 *versus* cells expressing wild-type BTN3A1 and mutated BTN3A2 ^41^ and unpublished data from the Morita group (personal communication) coming to the same conclusion by testing the response of a γδ T cell clone to Zoledronate-pulsed BTN3A knock out cells expressing V- and C-domain mutants of BTN3A1 and BTN3A2. It is further supported by the HMBPP-induced stimulation by 3KO cells expressing homomer-like BTN3A3-derivatives consisting of 3A3 and V-domain mutated super BTN3 (3A3 + 3A3_K136A_R381H) whose possible mechanistic basis will be discussed later.

Several aspects of the contribution of the JM of BTN3A to PAg stimulation were analyzed in previous studies. Firstly, PAg binding to the B30.2 domain was described and changes in the JM were found to be linked to PAg-induced stimulation ^29,30^. Vantourout and colleagues noted the importance of association of BTN3A1 and BTN3A2 molecules as well as the superiority of BTN3A1-BTN3A2 heteromers over BTN3A1 homomers in stimulation. Also identified were ER retention motifs in the JM of both molecules, which control intracellular trafficking and cell surface expression and are crucial for PAg-induced stimulation but could not explain the superiority of BTN3A heteromers over homomers ^33^. Finally, the Scotet group showed an increase in stimulation after replacing the JM of BTN3A1 with 3A3JM ^36^. Importantly, the current study can discriminate BTN3A complexes efficiently mediating PAg-stimulation from weak or non-stimulatory forms. It defines JM-controlled features: firstly, the rescue of surface expression of a paired V-deleted BTN3A molecule and secondly, in the case of BTN3A complexes, adaptation of a conformation that supports FRET between C-terminal fluorochromes. Notably, both cell surface rescue and efficient C-terminal FRET were not achieved for exclusively 3A1_JM containing molecules unless they were co-expressed with other BTN3A2 or BTN3A3 or 3A3_JM containing constructs. The high efficacy of heteromers that contain only a single PAg-binding site or in the case of BTN3A1-BTN3A2 dimers even only a single B30.2 domain over BTN3A1 homodimers is of special importance when discussing models postulating certain conformers of the extracellular domains (e.g. head to tail *versus* V-shaped dimers) or B30.2 domain dimers (symmetric *versus* asymmetric) ^29,44–47^ as being crucial for PAg-induced activation. Intriguingly, rescue of surface expression of VΔBTN3A1 as an indicator for successful formation of BTN3A-complexes coincided very well with molecular modeling results on forces determining stabilization of coiled coil structures formed by JM α-helices, which are reduced for 3A1 JM and favor interaction between BTN3A3 JM or alpaca BTN3JM, and heteromeric BTN3A1 JM interactions with BTN3A2 or BTN3A3JM. The residual activation seen with (overexpressed) BTN3A1 or 3A1JM containing constructs (Fig. 1a–d and 3a) might result from a small number of molecules still adopting a suitable extracellular BTN3A1-BTN2A1 topology despite unfavorable JM association ^29,33,34^

Our phylogeny informed approach to assign functions to certain BTN3A-regions allowed the identification of the 3A3_R381H mutant and a 3A1_3A3JM chimera as “super” BTN3A, merging the functions of heteromeric human BTN3A complexes in single, homomer-forming BTN3A molecules naturally occurring in the alpaca. The primordial BTN3A has been predicted to be a BTN3A3-like molecule with a functional PAg-binding site that emerged with placental mammals ^34,48,49^. This raises the question of what might have favored the evolution of BTN3A heteromers in primates ^27^ despite the efficacy of BTN3A homomers as witnessed in alpaca ^34^. Duplication of functional genes directly allows acquisition of new features even if these might have negative effects on the original function. This appears the case in humans, whereby the partnering BTN3A2 and BTN3A3 even lost PAg-binding function, which is compensated by formation of new functional units via heteromerization with BTN3A1, thereby preserving the *BTN3A-TRGV9-TRDV* triad mandatory for PAg-sensing. One possibility is that devolving from a single BTN3A molecule a substantial element of control of intracellular trafficking and IgV-related functionality may enable local fine-tuning of the strength of PAg-sensing via regulation of BTN3A2 and BTN3A3 expression. It will also be of interest to determine whether BTN3A1-JM might contribute to Vγ9Vδ2 T cell independent features of BTN3A1, including ligation of CD45 ^50^ or control of induction of type I interferon production by cytosolic TLR ligands ^51^.

Furthermore, it would be interesting to determine whether functional fusion proteins of different BTN relatives can also be achieved for the naturally occurring heteromers of Btn1/Btnl6, BTNL3/BTNL8, and Skint1/Skint2. Of note, such a fusion product is a frequently occurring copy number variation of *BTNL3* and *BTNL8*, resulting in fusion of intracellular BTNL3 with the BTNL8 extracellular domain ^52^ which would be expected not to bind Vγ4-TCR ^41^. This experiment of nature will allow testing of the physiological significance of the crosstalk, or the lack of it, between BTN(L) molecules, and to resolve the importance of TCR-BTNL3/8 binding for intestinal Vγ4 T-cell function, and gut homeostasis and pathophysiology ^53^. In addition, synthetic or natural “super” BTN3As such as that of alpaca might also be utilized as probes in the search for other factors involved in PAg-mediated Vγ9Vδ2 T cell activation.

A fourth key finding from our study was that we confirmed that HMBPP-binding to the BTN3A1 B30.2 domain promotes binding to the intracellular B30.2 domain of BTN2A1, and is consistent with our prior study ^20^ highlighting this interaction only occurs in the presence of a BTN3A1-B30.2-bound PAg such as HMBPP. Zhang group recently reported this interaction by size exclusion chromatography and an HMBPP coordinated complex consisting of an HMBPP-bound single BTN3A1-B30.2 domain and a dimer of BTN2A1 B30.2 domains ^21^. Notably, our ITC data are consistent with that model because we observe an n value near 1, which may be expected if a dimer of BTN2A1 is interacting with a monomeric PAg-ligand-bound form of BTN3A1-B30.2. The importance of PAg-induced interaction between BTN3A1-ID and- BTN2A1-ID for PAg-induced activation is also in line with that BTN3A1-B30.2 complexes with 4-M-HMBPP being a very poorly stimulatory analog of HMBPP ^44^, as it does not support this interaction.

**Figure 6:**
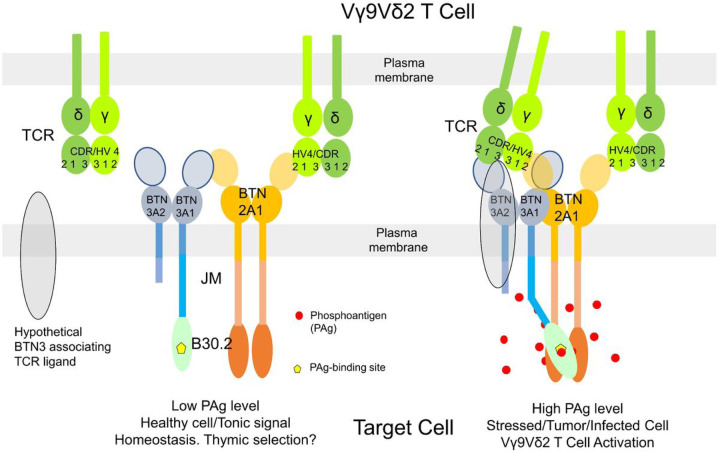
PAg induced Vγ9Vδ2 T cell activation by BTN3A-BTN2A1 composite ligand. In a resting state of the target cell, the heteromeric BTN3A (BTN3A1-BTN3A2/BTN3A3) interacts with BTN2A1 via their V-domains, and the BTN2A1-V domain interacts with germ-line encoded HV4 and CRR2 regions of Vγ9 chain of Vγ9Vδ2 TCR. Such interaction may act like a tonic TCR signal for maintaining homeostasis or even could be involved in the thymic selection of T cells. However, in case of stress in the target cell, the accumulated PAg binds to the B30.2 domain of BTN3A1, which further interacts with the B30.2 domains of BTN2A1. Consequently, the heteromeric JM region in the BTN3A complex permits the formation of appropriate topology where the V-domain of partnering BTN3A (BTN3A2/BTN3A3) distal to the PAg-B30.2 domain of BTN3A1, either on its own or in combination with unknown hypothetical ligand could be activating the TCR in which molecular interaction triggering remains elusive.

Based on these findings we formulate the following working hypothesis as a model (Fig. 6). PAg-binding to the BTN3A1-B30.2 domain renders the BTN3A1-HMBPP complex into a ligand for the BTN2A1 intracellular domain. The function of the BTN2A1-V domain would be to recruit the TCR by binding to the CDR2 and HV4 regions of the TCRγ chain, and that of BTN2A1 intracellular domain to recruit the HMBPP-bound BTN3A1-V. In the new complex, binding of TCRγ (CDR2 and HV4) chain to the C-F-G surface of BTN2A1-V domain would be retained, while other CDRs might additionally interact with the newly formed BTN2A1-BTN3A complex that is in line with the findings of Willcox research group. A direct interaction of the Vγ9Vδ2 TCR with V-domains of BTN2A1-BTN3A complexes would also be compatible with a most recent report that shows direct stimulation of Vγ9Vδ2 T cells by recombinant BTN3A1-BTN2A1 heteromers in the presence of a co-stimulus ^54^. However, it is yet to be proven whether BTN2A1 and BTN3A1 can form a functional heterodimer. In conclusion, our composite ligand model would allow inside-out signaling induced by conformational changes of the intracellular domains of BTN3A and BTN2A1 molecules without direct induction of conformational changes of their extracellular domains and predicts the formation of a new BTN2A1/BTN3A-TCR complex or BTN2A1/BTN3A plus hypothetical TCR-ligand - TCR complex in which both germline-encoded and somatically recombined CDRs of TCR chains are engaged. Such interactions are likely to surpass the requirements to initiate TCR signaling (Fig. 6).

The scenario discussed above is hypothetical and final clarification of the exact nature of the ligand recognized by the Vγ9Vδ2 TCR during PAg-activation has still to be elucidated. Nevertheless, the data we present and the molecular ground rules they formulate will be instrumental in guiding future studies to resolve this problem.

### Contact for Reagents and Resource Sharing

For further information and requests for reagents please contact the lead author (herrmann-t@vim.uni-wuerzburg.de)

### Experimental models and cell lines

53/4 hybridoma TCR transductants were cultured with RPMI (Gibco) supplemented with heat inactivated 10% FCS, 1 mM sodium pyruvate, 2.05 mM glutamine, 0.1 mM nonessential amino acids, 5 mM β-mercaptoethanol, penicillin (100 U/mL) and streptomycin (100 U/mL). Peripheral blood mononuclear cells were isolated from healthy volunteers. They were also maintained with the above-mentioned medium with or without rhIL-2 (Novartis Pharma). 293T cells were maintained in DMEM (Gibco) supplemented with 10% FCS.

## Method Details

### Generation of 293T BTN3AKO cells

293T BTN3KO (3KO) and BTN3A1KO (A1KO) cells used were mentioned in our previous study. The BTN3A2KO (A2KO), BTN3A3KO (A3KO) and BTN3A2 & BTN3A3KO (A2A3KO) cells were also generated as previously reported ^34^. The CRISPR sequences and the primers used for the validation of KO with genomic DNA are mentioned in the Supplementary Table 1.

### Generation of BTN3A, tagged BTN3A and BTN3A-fluorescent protein constructs

The full-length BTN3A1 and BTN3A1-mCherry fusion construct were generated as mentioned previously ^34^. The full-length BTN3A2 and BTN3A3 were subcloned from previously reported pIRES1hyg vectors ^15^. For the generation of pIH-FLAG, pIH vector ^55^ was digested with EcoRI and BamHI. Sequentially, the insert with Mfe1 and BglII restriction sequences as 5` and 3´ overhangs that comprises BTN3A1 leader sequence followed by FLAG sequence, linker sequence, and restriction sites for BamHI and EcoRI was digested with MfeI and BglII and cloned to EcoRI-BamHI digested pIH vector. This vector was further digested with BamHI and EcoRI and used to clone the desired BTN3A sequence from IgV to stop codon or IgC to stop (VΔ3A1 or VΔ3A2) sequence. pIZ-HA tagged BTN3A1 or BTN3A1_A3JM was generated with EcoRI and BamHI digested pIZ vector ^55^. Two PCR products with overlapping overhang sequences in which product 1, BTN3A1 leader sequence followed by HA tag and linker sequence (used above) and product 2, BTN3A1-IgV-domain till stop codon were cloned into above-digested pIZ vector using In-Fusion HD cloning (TAKARA) as per manufacturer’s instruction. The BTN3A1_A3JM chimera was subcloned from below mentioned pCDNA 3.1 vector. The multiple cloning site sequences pIH-FLAG and pZ-HA are provided in Table S1. GeneArt gene synthesis (ThermoFischer Scientific) synthesized the full-length BTN3A_JM chimeras by swapping the nucleic acids encoding for the JM region (272–340 amino acid ^36^ between BTN3A1 and BTN3A3. The JM chimeras cloned in pCDNA 3.1 vector were provided by the manufacturer and JM chimeras were further subcloned into phNGFR linker mCherry vector. phNGFR linker mCherry was used as the backbone to generate phNGFR linker CFP and phNGFR linker YFP, to which FLAG-3A1 or FLAG-VΔ3A1 and BTN3A1 or BTN3A1_A3JM chimera was subcloned, respectively. NEB 5-alpha (NEB) was used as transformant of the above-mentioned plasmids. The plasmids cloned with wild type BTN3A proteins or mutant BTN3A were expressed in 293T 3KO via retroviral transduction ^56^. All the restriction enzymes were purchased from Thermo Fischer Scientific. All the plasmids and cloned corresponding constructs were mentioned in Supplementary Table 2

### In vitro stimulation of human Vγ9Vδ2 TCR transductants

1*10^4^ 293T (DSMZ, ACC 635) or KO and their BTN3A transductants were seeded in 50 μL DMEM medium in 96 well flat-bottom tissue culture plate on day 1 and incubated overnight. On day 2, 50 μL of 53/4 r/mCD28 human Vγ9Vδ2 TCR transductants (MOP)^24^ at 1*10^6^ cells/mL density and 100 μL of HMBPP (SIGMA, 95058) at mentioned concentrations were added to the culture and incubated for 22 hours at 37°C. Post 22 hours, the activation of TCR reporter cells was measured by analyzing the supernatants of cocultures for mouse IL-2 via ELISA (Invitrogen, 88–7024-88) as per the manufacturer’s protocol.

### Expansion of primary polyclonal human Vγ9Vδ2 T cells

Fresh peripheral blood mononuclear cells (PBMCs) were obtained from healthy volunteers with informed consent according to the University of Wuerzburg institutional review board (Gz. 20220927 01). Tubes preloaded with Histopaque-1077 (SIGMA, 10711) were layered with whole blood and centrifuged at 400*g for 20 mins at room temperature with no acceleration or brakes. The opaque interface containing PBMCs was aspirated after centrifugation and was washed twice at 461*g for 5 mins. PBMCs were cultivated with RPMI containing heat inactivated 10% FCS, 100 IU/mL recombinant human IL-2 (Novartis Pharma) and 10 nM BrHBPP in 10^6^ cells/mL density in a 96 well plate round bottom plate. After 10 days, cells were pooled and washed twice, and cultured in a 6 well plate in 10^6^ cells/mL for 3 days without rhIL-2. Such rested cells were subjected to further experiments.

### Human polyclonal Vγ9Vδ2 T cell activation assay

293T cells at 2*10^4^ cells/100 μL (DMEM, 10% FCS) per well were cultured in triplicates in 96 well-plate flat bottom with or without 25 μM zoledronate (SIGMA) overnight. The next day, cells were washed twice with PBS, and Vγ9Vδ2 T cells expanded from PBMCs at 2*10^4^ cells/100 μL per well were added and cultured for 4 hours. After 4 hours, supernatants were frozen at −20°C until human INFγ assay ELISA (Invitrogen, EHIFNG) could be performed as per the manufacturer’s instructions. For the CD107a assay, 293T cells were seeded as above-mentioned. Vγ9Vδ2 T cells expanded from PBMCs were also added as above-mentioned but along with anti-CD107a-PE (BD Pharmingen) conjugated antibody and cultured for 4 hours. After 4 hours, the cells were collected from the wells as triplicates and washed once with PBS. After which cells were treated with anti-human Vδ2-FITC (Beckman Coulter) conjugated antibody for 20 mins and washed once, followed by analysis at FACSCalibur (BD) for the percentage of Vδ2-FITC and CD107a-PE population.

### Flow cytometry for surface and total expression of BTN3As

293T and 3KO transductants of BTN3As (WT and Chimaeras) were acquired by FACScalibur (BD) and analyzed with FlowJo. For total staining, cells were fixed with fixation buffer for 30 mins at RT, followed by wash and incubated for 30 mins with permeabilization buffer at RT. Then cells were stained with antibodies that were prediluted in permeabilization for 30 mins at 4°C, as per the manufacturer’s instructions (eBiosciences, eBiosciences^™^ Intracellular Fixation & Permeabilization buffer set). For surface staining, cells were directly stained with antibodies of interest for 30 minutes at 4°C. The BTN3As were detected by unconjugated mAb 103.2 (gift from Daniel Olive). If tagged, unconjugated anti-FLAG (M2, SIGMA) and anti-HA (F-7, Santa Cruz) antibodies were used. The primary antibodies were detected by Fab Donkey anti mouse IgG (H + L)-APC (Jackson Immunoresearch, 115–136-146). mIgG1k and mIgG2a k (eBiosciences) were used as isotype controls.

### Immunoprecipitation

3*10^6^ cells of 3KO and BTN3A-transductants were seeded in a 10 cm tissue culture plate on day 1. On day 3, the cells were lysed with 400 μL of lysis buffer ^33^ [(50 mM Tris·HCl at pH 7.4, 150 mM KCl, 10 mM MgCl_2_, 1 mM CaCl_2_, 0.5% Nonidet P-40, 0.1% digitonin, 5% glycerol, Complete Protease inhibitor(Roche)]. The lysate was rigorously vortexed for 15 mins at 4°C and was centrifuged at 14,000 rpm for 15 mins at 4°C. After centrifugation, 50 μL lysate was kept aside as input. The remaining lysate was incubated for 4 hours at 4°C with 50 μL of protein-G Sepharose^™^ (GE, 1706180) beads complexed with anti-FLAG (M2 clone, SIGMA) and washed thrice with lysis buffer. Proteins were eluted with 80 μL of Laemmli and analyzed by SDS-PAGE and Western Blotting. The blots were treated with anti-Vinculin (SIGMA), anti-FLAG and anti-HA (CST) as primary antibodies overnight at 4°C. The following day, the blots were washed thrice and treated with protein-A-HRP (SIGMA) conjugate for an hour at RT and washed and developed with Pierce SuperSignal^™^ West Femto Maximum Sensitivity Substrate (Thermo Fischer Scientific). The blots were visualized with LI-COR Odyssey imaging system.

### Blue native gel electrophoresis

Blue native gel electrophoresis was performed as described in ^57^.

### Immunofluorescent staining

293T, 3KO and 3KO-BTN3A transductants were seeded in 5*10^4^/200 μL in Ibidi 8 well μSlides on day 1. On day 2, for live-cell imaging, cells were washed twice with PBS and treated with anti-FLAG (M2) or anti-HA for 20 mins, followed by three washes and treated with anti-mouse AF648 (Invitrogen) or anti-Rabbit AF565 (Invitrogen) for 30 mins. After 30 mins, cells were washed thrice and visualized with confocal microscope Zeiss LSM 780 under 63x (NA 1.4) oil immersion lens with 514 and 633 lasers. Acquired images were further analyzed using ImageJ. For fixed cell imaging, the cells were fixed with 4% paraformaldehyde for 30 mins and either treated with 0.1% TritonX-100 for permeabilization or treated with anti-FLAG or anti-HA antibodies overnight. The following day, cells were washed and treated with anti-mouse AF648 or anti-rabbit AF565 for 1 hour and washed thrice before acquiring images under the microscope as above.

### Fluorescence recovery after photobleaching

293T and 3KO transduced with BTN3A1-mCherry fusion construct were seeded in Ibidi 8well μSlides at 5*10^4^/200 μL per well on day 1. On day 2 cells were analyzed with confocal microscope Zeiss LSM 780 under a 63x (NA 1.4) oil immersion lens with a 560 laser. The rectangular regions were marked on the cells of interest, the marked regions were photobleached with 100% laser energy for 5 seconds (> 90% loss of fluorescence). Images were collected after every 5 seconds after photobleaching for 100 seconds. The percentage of the immobile fraction was derived from the below-mentioned formula

Mobile fraction F_m_ = (I_E_ - I_0_) / (I_I_ - I_0_); Immobile fraction F_i_ = 1 – Fm; where: I_E_: Endvalue of the recovered fluorescence intensity, I_0_: first postbleach fluorescence intensity, I_I_: Initial (prebleach) fluorescence intensity.

### Fluorescence resonance energy transfer

3KO transduced with FLAG-BTN3A1-CFP or FLAG-VΔ3A1-CFP and BTN3A1-YFP or BTN3A1_A3JM YFP constructs were plated over the glass coverslips. Before imaging, cells were incubated in the imaging medium (144 mM NaCl, 5.4 mM KCl, 1 mM MgCl2, 1 mM CaCl2, 10 mM HEPES; pH = 7.4) and mounted on Leica DMI 3000 B microscope fitted with a 63x/1.40 objective. The cells were excited with CoolLED (440 nm) and the emission light was split into donor and acceptor channels using the DV2 QuadView (Photometrics) equipped with the 505dcxr dichroic mirror and D480/30m and D535/40m emission filters. When CFP and YFP are in FRETable distance, the emitted light detected by 535 filters (YFP) would be greater than 480 filters which can be presented as pseudo-colored ratio images with a reference FRET ratio (FR) chart.Images were acquired using CMOS camera (OptiMOS, QImaging) and MicroManager 1.4. software was used for data analysis^58,59^.

### Synthesis of 4-M-HMBPP

Binding of 4-hydroxy-3-(4-methylbenzyl)but-2-en-1-yl diphosphate (4-M-HMBPP) to BTN3A1 was previously described by Yang et al. ^44^ but the synthetic route has not yet been reported. We adapted the method of Yang et al. (Yonghui Zhang, personal communication to TH) to obtain 4-M-HMBPP as detailed in the supplemental for use in these studies.

### Isothermal titration calorimetry (ITC):

ITC was performed as described ^20^ using a nanoITC (TA Instruments). The concentrations of the titrant and titrand are indicated in the figure legend.

### Modelling BTN3 juxtamembrane coiled-coil dimers

Models of the juxtamembrane (JM) coiled-coil dimers were generated using the CCBuilder2 server (http://coiledcoils.chm.bris.ac.uk/ccbuilder2/builder) ^43^. Models were generated using default settings assuming a parallel homo/hetero dimeric structure, encompassing residues Q273–L312 for human BTN3A1, BTN3A2, and BTN3A3 and alpaca BTN3A3. BTN3A1 was modelled with Q273 at the “c” position of the heptad repeat, whereas all other BTN3 molecules were modelled with Q273 at the “d” position. Models of human BTN3 proteins were further refined using the “Optimize” function of the CCBuilder2 program. JM coiled-coil dimer interface contacts were determined using the program NCONT as part of the CCP4 suite ^60^. Structural figures were generated using PyMol ^61^.

### Statistics

Statistical analysis of stimulations was performed with GraphPad Prism using two-way ANOVA and statistical significance in terms of P-value adjusted as per GraphPad Prism tool are presented in asterisk (*) (**** <0.0001; *** <0.001; ** <0.01; * <0.05; ns > 0.05). Similarly, samples analyzed for FRAP were subjected to multiple t-tests and statistical significance was determined using the Bonferroni-Dunn method. The representation of statistical significance in P-value as asterisks (*) or non-significant (ns) as above was adjusted in GraphPad Prism.

## Figures and Tables

**Table 1:** 

Titrant	Titrand	K_D_(μM)	n	ΔH (kJ/mol)	ΔS (J/mol*K)
BTN2A1 ID271	BTN3A1 BFI + HMBPP	0.78 ± 0.28	0.94 ± 0.08	−48.66 ± 2.11	−45.62 ± 7.54
BTN2A1 ID271	BTN3A1 BFI + 4-M-HMBPP	189.9 ± 174.8	0.08 ± 0.06	−100 ± 0	−260.4 ± 7.92

a The binding parameters are obtained by independent fit using NanoAnalyze. Dates represent the mean ± SEM. (n = 3 independent experiments).
